# Supplementary material for: Protection against symptomatic dengue infection by neutralizing antibodies varies by infection history and infecting serotype
Source: Nat Commun. 2024 Jan 9;15:382. doi: 10.1038/s41467-023-44330-8 (PMC10776616; doi:10.1038/s41467-023-44330-8)
Supplement: Supplementary file 1 — Supplementary Information [file 41467_2023_44330_MOESM1_ESM.pdf]

# Figure S1

A.

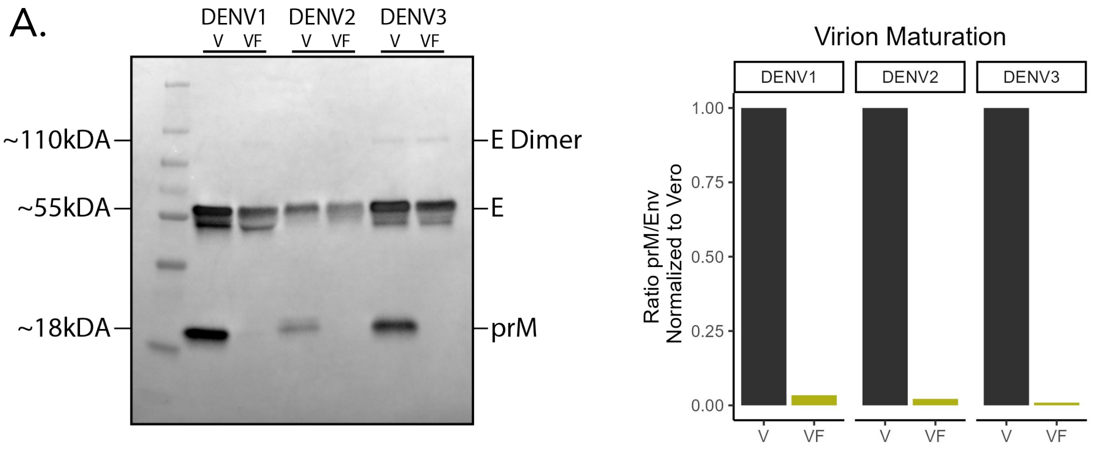


B.

| | | | Vero | | Vero DC-SIGN | |
|---|---|---|---|---|---|---|
| | | Copy genome (vRNA/mL) | Viral Titer (FFU/mL) | Infectivity (vRNA:FFU) | Viral Titer (FFU/mL) | Infectivity (vRNA:FFU) |
| DENV1 | Partially Mature | 5.55E+09 | 2.70E+06 | 2057 | 9.60E+06 | 578 |
| | Mature | 2.45E+09 | 4.30E+06 | 569 | 5.90E+06 | 415 |
| DENV2 | Partially Mature | 2.42E+10 | 1.13E+06 | 21386 | 1.90E+06 | 12719 |
| | Mature | 1.02E+10 | 1.58E+06 | 6428 | 3.30E+06 | 3078 |
| DENV3 | Partially Mature | 9.33E+09 | 7.87E+05 | 11859 | 2.73E+06 | 3417 |
| | Mature | 7.14E+09 | 1.92E+06 | 3729 | 6.43E+06 | 1112 |

C. D.

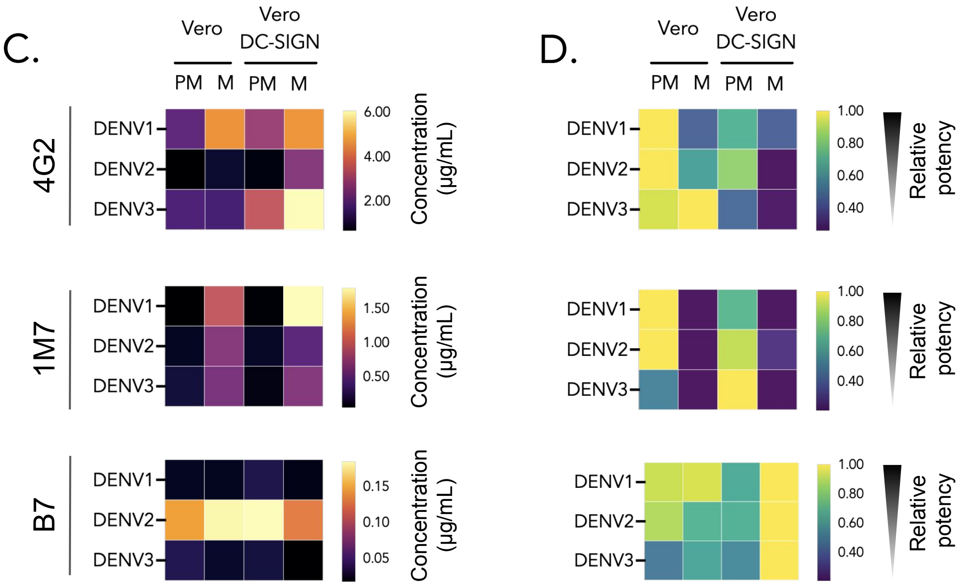

**Figure S1. Characterization of maturation state, infectivity and mAb neutralization of viral stocks.** (A) Western blot image (left) of DENV1-3 viral supernatants from Vero (V) and Vero-furin cells (VF) stained with anti-E and anti-prM antibodies. Each viral stock was first tested individually and then side by side in the western blot presented. (B) Quantification of the increase in maturation state (prM/E) of DENV1-3 virions produced in VF cells compared to Vero cells (lower value = more mature) as seen by relative western blot band intensity. (C) Neutralization potency of monoclonal antibodies (mAbs), with $EC_{50}$ values in ug/mL displayed across assay conditions. The darker color corresponds to mAbs with the highest neutralization potency. (D) Relative neutralization potency of mAbs. Values are normalized by the lowest $EC_{50}$ observed among the four assay conditions (most potent), calculated as (1 / ($EC_{50}$ of assay condition X / lowest $EC_{50}$ of four assay condition)). Large color variation indicates a strong impact of change in assay conditions. Relative $EC_{50}$ value of 1 (yellow) indicates highest potency, of 0.2 (dark purple) least potent. Source Data are provided as a Source Data file.

**Validation of mAb used in the figure :**

- Anti-pr mAbs: 2G3, 1E23, and 2H21 were validated by Smith SA, et al. 2015. Dengue Virus prM-Specific Human Monoclonal Antibodies with Virus Replication-Enhancing Properties Recognize a Single Immunodominant Antigenic Site. J Virol. 2015 Oct 28;90(2):780-9. doi: 10.1128/JVI.01805-15. PMID: 26512092; PMCID: PMC4702676.
- mAb 1M7 was validated by Smith SA, et al. 2013. Human monoclonal antibodies derived from memory B cells following live attenuated dengue virus vaccination or natural infection exhibit similar characteristics. J Infect Dis 207:1898–1908. doi: 10.1093/infdis/jit119.
- mAb B7 was validated by Dejnirattisai W, et al. 2014. A new class of highly potent, broadly neutralizing antibodies isolated from viremic patients infected with dengue virus. Nat Immunol. 2015 Feb;16(2):170-177. doi: 10.1038/ni.3058. Epub 2014 Dec 15. PMID: 25501631; PMCID: PMC4445969.

Figure S2

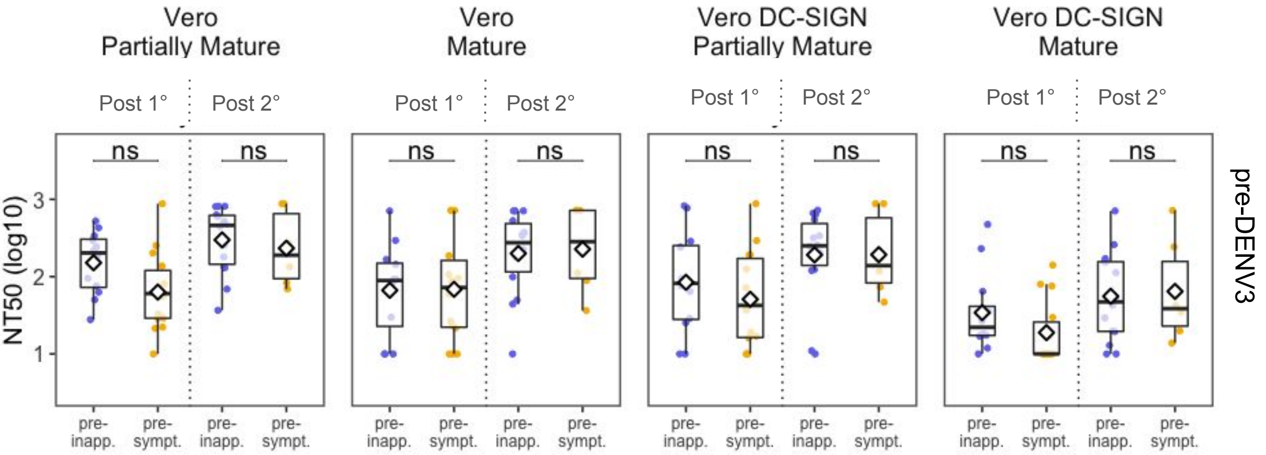


**Figure S2. Cross-reactive nAb titers of pre-inapparent and pre-symptomatic DENV3 infection participants stratified by infection history.** Shown are median NT50 (middle line), 25th to 75th percentile (box), and 5th to 95th percentile (whiskers) as well as the raw data (points). Diamond indicates mean NT50. Asterisks indicate Benjamini Hochberg adjusted p-values for Wilcoxon test (two-sided). p-values: ns, >0.05; *, <0.05; **, <0.01; ***, <0.001; ****, <0.0001. Post-1°: post-primary infection, Post-2°: post-secondary infection. pre-inapp. = pre-inapparent DENV infection group (purple), pre-sympt. = pre-symptomatic DENV infection group (yellow).

Sample size : post 1° pre-inapp n= 12, post 1° pre-sympt n= 14, post 2° pre-inapp n= 14, post 2° pre-sympt n= 6.

**Table S1. ROC analysis and logistic regression of $NT_{50}$ magnitude as a predictor for developing a subsequent symptomatic disease, stratified by infection history and incoming serotype**.

| | Combined | | | | | | |
|---|---|---|---|---|---|---|---|
| | AUC[a] | CI 95[b] | Threshold ($NT_{50}$) | Sensitivity | Specificity | OR[c] | CI 95 |
| **DENV1** | | | | | | | |
| Part.Mature[d] Vero | 0.74 | 0.50 to 0.99 | 325.90 | 1 | 0.44 | | *ns* |
| Mature Vero | 0.77 | 0.51 to 1 | 206.84 | 1 | 0.67 | | *ns* |
| Part.Mature[d] Vero DC-SIGN | 0.82 | 0.61 to 1 | 227.00 | 1 | 0.44 | | *ns* |
| Mature Vero DC-SIGN | 0.68 | 0.42 to 0.94 | 148.35 | 0.9 | 0.44 | | *ns* |
| **DENV2** | | | | | | | |
| Part.Mature[d] Vero | 0.62 | 0.47 to 0.77 | 968.34 | 1 | 0.18 | 0.86 | 0.73 to 0.99 |
| Mature Vero | 0.71 | 0.57 to 0.85 | 441.75 | 0.91 | 0.5 | 0.69 | 0.52 to 0.85 |
| Part.Mature[d] Vero DC-SIGN | 0.65 | 0.50 to 0.79 | 714.55 | 0.94 | 0.21 | 0.83 | 0.68 to 0.98 |
| Mature Vero DC-SIGN | 0.68 | 0.54 to 0.82 | 229.85 | 0.91 | 0.32 | 0.64 | 0.41 to 0.9 |
| **DENV3** | | | | | | | |
| Part.Mature[d] Vero | 0.52 | 0.52 to 0.85 | 281.35 | 0.85 | 0.46 | | *ns* |
| Mature Vero | 0.51 | 0.33 to 0.70 | 201.10 | 0.7 | 0.42 | | *ns* |
| Part.Mature[d] Vero DC-SIGN | 0.61 | 0.44 to 0.78 | 304.75 | 0.85 | 0.31 | | *ns* |
| Mature Vero DC-SIGN | 0.64 | 0.47 to 0.81 | 148.10 | 0.9 | 0.27 | | *ns* |

| | Primary | | | | | | |
|---|---|---|---|---|---|---|---|
| | AUC[a] | CI 95[b] | Threshold ($NT_{50}$) | Sensitivity | Specificity | OR[c] | CI 95 |
| **DENV1** | | | | | | | |
| Part.Mature[d] Vero | 0.74 | 0.50 to 0.99 | 325.90 | 1 | 0.44 | *ns* | - |
| Mature Vero | 0.77 | 0.51 to 1 | 206.84 | 1 | 0.67 | *ns* | - |
| Part.Mature[d] Vero DC-SIGN | 0.82 | 0.61 to 1 | 227.00 | 1 | 0.44 | *ns* | - |
| Mature Vero DC-SIGN | 0.68 | 0.42 to 0.94 | 148.35 | 0.9 | 0.44 | *ns* | - |
| **DENV2** | | | | | | | |
| Part.Mature[d] Vero | 0.65 | 0.42 to 0.87 | 558.15 | 1 | 0.38 | *ns* | - |
| Mature Vero | 0.72 | 0.51 to 0.93 | 465.65 | 1 | 0.46 | 0.62 | 0.38 to 0.88 |
| Part.Mature[d] Vero DC-SIGN | 0.70 | 0.50 to 0.90 | 351.50 | 0.94 | 0.38 | *ns* | - |
| Mature Vero DC-SIGN | 0.75 | 0.56 to 0.95 | 74.88 | 0.94 | 0.54 | 0.09 | 0.01 to 0.52 |
| **DENV3** | | | | | | | |
| Part.Mature[d] Vero | 0.73 | 0.52 to 0.93 | 273.55 | 0.93 | 0.33 | *ns* | - |
| Mature Vero | 0.49 | 0.26 to 0.72 | 240.10 | 0.79 | 0.17 | *ns* | - |
| Part.Mature[d] Vero DC-SIGN | 0.59 | 0.37 to 0.82 | 532.60 | 0.93 | 0.17 | *ns* | - |
| Mature Vero DC-SIGN | 0.73 | 0.52 to 0.93 | 110.55 | 0.93 | 0.17 | *ns* | - |

| | Secondary | | | | | | |
|---|---|---|---|---|---|---|---|
| | AUC[a] | CI 95[b] | Threshold ($NT_{50}$) | Sensitivity | Specificity | OR[c] | CI 95 |
| **DENV1** | | | | | | | |
| Part.Mature[d] Vero | - | - | - | - | - | - | - |
| Mature Vero | - | - | - | - | - | - | - |
| Part.Mature[d] Vero DC-SIGN | - | - | - | - | - | - | - |
| Mature Vero DC-SIGN | - | - | - | - | - | - | - |
| **DENV2** | | | | | | | |
| Part.Mature[d] Vero | 0.60 | 0.38 to 0.81 | 981.84 | 1 | 0.2 | *ns* | |
| Mature Vero | 0.68 | 0.48 to 0.89 | 475.83 | 1 | 0.47 | 0.72 | 0.5 to 0.95 |
| Part.Mature[d] Vero DC-SIGN | 0.61 | 0.4 to 0.83 | 853.75 | 0.94 | 0.27 | *ns* | |
| Mature Vero DC-SIGN | 0.66 | 0.45 to 0.87 | 455.50 | 0.94 | 0.33 | *ns* | |
| **DENV3** | | | | | | | |
| Part.Mature[d] Vero | 0.51 | 0.16 to 0.86 | 810.73 | 0.67 | 0.14 | *ns* | - |
| Mature Vero | 0.59 | 0.23 to 0.97 | 23.18 | 1 | 0.07 | *ns* | - |
| Part.Mature[d] Vero DC-SIGN | 0.55 | 0.18 to 0.91 | 693.55 | 0.67 | 0.07 | *ns* | - |
| Mature Vero DC-SIGN | 0.44 | 0.15 to 0.74 | 483.97 | 0.83 | 0.07 | *ns* | - |

[a]AUC = Area under the curve, [b]CI95 = 95% Confidence interval, [c]OR = Odds ratio, [d]Part. Mature = Partially mature virion.
