# Supplementary material for: Protection against symptomatic dengue infection by neutralizing antibodies varies by infection history and infecting serotype
Source: Nat Commun. 2024 Jan 9;15:382. doi: 10.1038/s41467-023-44330-8 (PMC10776616; doi:10.1038/s41467-023-44330-8)
Supplement: Supplementary file 3 — Reporting Summary [file 41467_2023_44330_MOESM3_ESM.pdf]

nature portfolio

Corresponding author(s): Eva Harris, Sandra Bos

Last updated by author(s): Nov 27, 2023

Reporting Summary

Nature Portfolio wishes to improve the reproducibility of the work that we publish. This form provides structure for consistency and transparency in reporting. For further information on Nature Portfolio policies, see our Editorial Policies and the Editorial Policy Checklist.

Statistics

For all statistical analyses, confirm that the following items are present in the figure legend, table legend, main text, or Methods section.

n/a	Confirmed	
☐	☒	The exact sample size (*n*) for each experimental group/condition, given as a discrete number and unit of measurement
☐	☒	A statement on whether measurements were taken from distinct samples or whether the same sample was measured repeatedly
☐	☒	The statistical test(s) used AND whether they are one- or two-sided<br>*Only common tests should be described solely by name; describe more complex techniques in the Methods section.*
☒	☐	A description of all covariates tested
☐	☒	A description of any assumptions or corrections, such as tests of normality and adjustment for multiple comparisons
☐	☒	A full description of the statistical parameters including central tendency (e.g. means) or other basic estimates (e.g. regression coefficient) AND variation (e.g. standard deviation) or associated estimates of uncertainty (e.g. confidence intervals)
☐	☒	For null hypothesis testing, the test statistic (e.g. *F*, *t*, *r*) with confidence intervals, effect sizes, degrees of freedom and *P* value noted<br>*Give P values as exact values whenever suitable.*
☒	☐	For Bayesian analysis, information on the choice of priors and Markov chain Monte Carlo settings
☒	☐	For hierarchical and complex designs, identification of the appropriate level for tests and full reporting of outcomes
☒	☐	Estimates of effect sizes (e.g. Cohen's *d*, Pearson's *r*), indicating how they were calculated

Our web collection on statistics for biologists contains articles on many of the points above.

Software and code

Policy information about availability of computer code

Data collection	CTL Immunospot and GraphPad PRISM Version 10 for Windows
Data analysis	R Studio was utilized for data analysis no custom algorithm was used. the following packages were used : readr 2.1.4, dplyr 1.1.1, ggplot2 3.4.2, rstatix 0.7.2, ggpubr 0.6.0, tableone 0.13.2, pROC 1.18.0, stats 4.2.2, oddsratio 2.0.1, pwr 1.3-0, pscl 1.5.5

For manuscripts utilizing custom algorithms or software that are central to the research but not yet described in published literature, software must be made available to editors and reviewers. We strongly encourage code deposition in a community repository (e.g. GitHub). See the Nature Portfolio guidelines for submitting code & software for further information.

Data

Policy information about availability of data

All manuscripts must include a data availability statement. This statement should provide the following information, where applicable:

- Accession codes, unique identifiers, or web links for publicly available datasets
- A description of any restrictions on data availability
- For clinical datasets or third party data, please ensure that the statement adheres to our policy

All data produced in the present study are available in the Source Data file provided with this paper. Data analysis was performed using R and packages that are publicly available, no custom code was generated. Any transfer of materials needs to be approved by the IRB and by Dr. Harris and Nicaraguan researchers. Further, the samples in question are from pediatric participants in our long-term cohort study and are therefore very limited in volume. Thus, these materials are subject to

restriction based on limited availability. For more information, please contact Dr. Eva Harris (eharris@berkeley.edu) and/or the committee for the Protection of Human Subjects at University of California Berkeley (510-642-7461; ophs@berkeley.edu).

Research involving human participants, their data, or biological material

Policy information about studies with human participants or human data. See also policy information about sex, gender (identity/presentation), and sexual orientation and race, ethnicity and racism.

Reporting on sex and gender	Sex- and gender-based analyses were not performed in this study as this parameter is not relevant to the study of neutralizing antibody titer magnitude in the pediatric population. Nevertheless, the sex ratio was balanced in our groups as described in tables 1 and 2.
Reporting on race, ethnicity, or other socially relevant groupings	No variables utilizing race, ethnicity, or other socially relevant categorization were used in this manuscript for purposes of study design.
Population characteristics	All participants live in District II, Managua, Nicaragua, the catchment area for the Health Center Socrates Flores Vivas (HCSFV). The age of study participants was limited to children ages 2 to 17 years old, as children have the highest burden of dengue in Nicaragua.
Recruitment	The Pediatric Dengue Cohort Study (PDCS) is an ongoing open prospective cohort of ~4,000 active children 2-17 years old that was initiated in 2004, in Nicaragua (Managua). To recruit cohort participants, in August and September of 2004, study teams performed house-to-house visits in neighborhoods served by the HCSFV and invited all eligible children to participate. Eligibility criteria included the following: 1) age between 2 and 9 years, 2) residence in the study area, 3) no plan to leave the study area during the following 3 years, 4) willingness to attend the study clinic for all medical needs, 5) no immune-compromising conditions, such as current chemotherapy treatment or human immunodeficiency virus positivity, 6) informed parental consent, and 7) participant assent for children over 5 years of age. To maintain the original age distribution of the cohort, ~300 two-year-olds, along with some children aged 3-11 years, are enrolled in the study each year. The age limit of the study was increased to 15, 16 in 2018, 2019, respectively. Blood samples are collected annually, and additional samples are collected from symptomatic suspected dengue cases at the acute (0-6 days post-onset of symptoms) and convalescent (14-21 days post-onset of symptoms) phase. Participants are provided free medical care 24 hours per day, 365 days per year, through study physicians at the HCSFV. The PDCS is described here : https://doi.org/10.1093/aje/kwp092.
Ethics oversight	The human subjects protocol for the PDCS was reviewed and approved by the Institutional Review Boards (IRB) of the University of California, Berkeley (2010-09-2245), the University of Michigan (HUM00091606), and the Nicaraguan Ministry of Health (CIRE-09/03/07-008).

Note that full information on the approval of the study protocol must also be provided in the manuscript.

Field-specific reporting

Please select the one below that is the best fit for your research. If you are not sure, read the appropriate sections before making your selection.

☒ Life sciences ☐ Behavioural & social sciences ☐ Ecological, evolutionary & environmental sciences

For a reference copy of the document with all sections, see nature.com/documents/nr-reporting-summary-flat.pdf

Life sciences study design

All studies must disclose on these points even when the disclosure is negative.

Sample size	Given that this study is nested on the Pediatric Dengue Cohort in Nicaragua and that statistical inference is limited by the epidemiological behavior of dengue in the area of study there we did not pre-defined a sample size. Instead, we selected a subset of 125 DENV-immune children who developed an inapparent infection (n=63) or symptomatic infection (n=62), matched by incoming serotype. Participant selection was performed as mentionned in "Randomization" section. Finally, we performed a post-hoc analysis (discussed in the discussion section) to establish the power of our design for detecting a significant difference in the neutralizing potency of the antibodies, measured using the assay conditions described in the Methods section, comparing the different clinical groups. Power calculations were conducted using the pwr package v. 1.3-0, and the package pscl v 1.5.5 was used for estimating the McFadden's pseudo R-squared of each model.
Data exclusions	No data were excluded from the analysis.
Replication	All serum samples were run in duplicate and replicated only if they did not pass quality control (R^2 > 0.8 and Hill slope > 0.5) for the neutralization assay. The western blot, demonstrating virion maturation, was conducted twice. Initially, each viral stock (i.e., serotype) was tested independently; subsequently, they were tested side by side in the second round.
Randomization	The criteria for sample selection were : 1) symptomatic infection: an annual sample from a child post-primary or post-secondary DENV infection collected prior to a subsequent DENV infection. Hence, individuals had been exposed in the past to other DENV serotypes and samples were collected at a time-point that is months or years after a previous infection, as well as months or years before a subsequent infection. 2) For inapparent infection: an annual sample from a child post-primary or post-secondary DENV infection collected prior to a subsequent DENV infection and right after the given infection in order to confirm the infecting serotype using neutralization assays of Luminex assay detecting type specific anti-DENV IgG. Inapparent infections detected in 2016-2019 were attributed to DENV2 based on laboratory and epidemiological data showing the circulation of only DENV2 in Nicaragua during these years.

	Group assignment was performed according to disease manifestation (symptomatic or inapparent infection), prior infection history (post-primary or post-secondary DENV infection), and infecting serotype (DENV1, DENV2 or DENV3)<br><br>For the pre-DENV2 subset (n=60), the sample selection was randomized balancing by infection outcome and prior infection history and infecting serotype. For the pre-DENV1 and DENV3 sample sets we included all the samples available at the time of the study, balancing by infection outcome and prior infection history as much as possible, and for which we could confirm the incoming serotype using the serological assays mentioned above (DENV1 n=19, DENV3 = 46).
Blinding	Blinding was not relevant to our study because it was necessary to understand what the incoming serotype was for the subsequent DENV infection. However, the disease outcomes were not known by the person performing the experiment.

Behavioural & social sciences study design

All studies must disclose on these points even when the disclosure is negative.

Study description	n/a
Research sample	n/a
Sampling strategy	n/a
Data collection	n/a
Timing	n/a
Data exclusions	n/a
Non-participation	n/a
Randomization	n/a

Ecological, evolutionary & environmental sciences study design

All studies must disclose on these points even when the disclosure is negative.

Study description	n/a
Research sample	n/a
Sampling strategy	n/a
Data collection	n/a
Timing and spatial scale	n/a
Data exclusions	n/a
Reproducibility	n/a
Randomization	n/a
Blinding	n/a

Did the study involve field work? ☐ Yes ☒ No

Field work, collection and transport

Field conditions	n/a
Location	n/a
Access & import/export	n/a
Disturbance	n/a

Reporting for specific materials, systems and methods

We require information from authors about some types of materials, experimental systems and methods used in many studies. Here, indicate whether each material, system or method listed is relevant to your study. If you are not sure if a list item applies to your research, read the appropriate section before selecting a response.

Materials & experimental systems

n/a	Involved in the study
☐	☒ Antibodies
☐	☒ Eukaryotic cell lines
☒	☐ Palaeontology and archaeology
☒	☐ Animals and other organisms
☐	☒ Clinical data
☒	☐ Dual use research of concern
☒	☐ Plants

Methods

n/a	Involved in the study
☒	☐ ChIP-seq
☒	☐ Flow cytometry
☒	☐ MRI-based neuroimaging

Antibodies

Antibodies used

4G2 monoclonal antibody (mAb)-mouse anti-FL epitope of the DENV envelope (E) protein. Supplier: Biomatik. Catalog #AB00230-2.0 Clone D1-4G2-15 Lot#T1619A11
mAb 1M7 human mAb anti-FL epitope of the DENV E protein. Obtained from Aravinda de Silva, University of North Carolina (UNC), Chapel Hill.
mAb B7 anti-Envelope dimer epitope of flaviviruses. Obtained from A. de Silva, UNC.
mAb 2G3 anti-pr peptide of DENV. Obtained from A. de Silva, UNC. 0.1ug/ml
mAb 1E23 anti-pr peptide of DENV. Obtained from A. de Silva, UNC. 0.1ug/ml
mAb 2H21 anti-pr peptide of DENV. Obtained from A. de Silva, UNC. 0.1ug/ml
Goat anti-mouse IgG Secondary antibody conjugated to HRP (1:3000, Biolegend, 405306)
Donkey anti-human IgG Secondary antibody conjugated to HRP (1:5000, Biolegend, 410902)
anti-CD209 (1:1000, BD Bioscience, BDB561765)

Validation

Commercial antibodies were validated by the company provider. Anti-pr mAbs: 2G3, 1E23, and 2H21 were validated by Smith SA, Nivarthi UK, de Alwis R, Kose N, Sapparapu G, Bombardi R, Kahle KM, Pfaff JM, Lieberman S, Doranz BJ, de Silva AM, Crowe JE Jr. Dengue Virus prM-Specific Human Monoclonal Antibodies with Virus Replication-Enhancing Properties Recognize a Single Immunodominant Antigenic Site. J Virol. 2015 Oct 28;90(2):780-9. doi: 10.1128/JVI.01805-15. PMID: 26512092; PMCID: PMC4702676. mAb 1M7 was validated by Smith SA, de Alwis R, Kose N, Durbin AP, Whitehead SS, de Silva AM, Crowe JE Jr. 2013. Human monoclonal antibodies derived from memory B cells following live attenuated dengue virus vaccination or natural infection exhibit similar characteristics. J Infect Dis 207:1898–1908. doi: 10.1093/infdis/jit119. mAb B7 was validated by Dejnirattisai W, Wongwiwat W, Supasa S, Zhang X, Dai X, Rouvinski A, Jumnainsong A, Edwards C, Quyen NTH, Duangchinda T, Grimes JM, Tsai WY, Lai CY, Wang WK, Malasit P, Farrar J, Simmons CP, Zhou ZH, Rey FA, Mongkolsapaya J, Screaton GR. A new class of highly potent, broadly neutralizing antibodies isolated from viremic patients infected with dengue virus. Nat Immunol. 2015 Feb;16(2):170-177. doi: 10.1038/ni.3058. Epub 2014 Dec 15. PMID: 25501631; PMCID: PMC4445969.

Eukaryotic cell lines

Policy information about cell lines and Sex and Gender in Research

Cell line source(s)

Vero 81 Cells were obtained from ATCC via the UC Berkeley cell culture facility. Vero cells were transduced using lentivirus to express DC-SIGN (CD209)

Authentication

Vero 81 Cells came authenticated from ATCC (human cell authentication assay identifies short tandem repeat (STR) markers at specific loci to establish a DNA fingerprint for every human cell line). DC-SIGN surface expression was confirmed by flow cytometry using a PE-conjugated mouse anti-CD209 antibody (BD Bioscience) for cell surface staining.

Mycoplasma contamination

Cells were cerfitied as mycoplasma-free from the UC Berekely Cell Culture Facility upon receipt. Cells in the Harris lab are routinely screened for mycoplasma.

Commonly misidentified lines
(See ICLAC register)

None

Palaeontology and Archaeology

Specimen provenance

n/a

Specimen deposition	n/a
Dating methods	n/a

☐ Tick this box to confirm that the raw and calibrated dates are available in the paper or in Supplementary Information.

Ethics oversight	n/a

Note that full information on the approval of the study protocol must also be provided in the manuscript.

Animals and other research organisms

Policy information about studies involving animals; ARRIVE guidelines recommended for reporting animal research, and Sex and Gender in Research

Laboratory animals	n/a
Wild animals	n/a
Reporting on sex	n/a
Field-collected samples	n/a
Ethics oversight	n/a

Note that full information on the approval of the study protocol must also be provided in the manuscript.

Clinical data

Policy information about clinical studies

All manuscripts should comply with the ICMJEguidelines for publication of clinical research and a completedCONSORT checklist must be included with all submissions.

Clinical trial registration	Protocol # 2010-09-2245
Study protocol	Study Protocol is available by request, as is required by the Institutional Review Board-approved protocol for the Pediatric Dengue Cohort Study. Please contact Dr. Eva Harris and/or the committee for the Protection of Human Subjects at University of California, Berkeley.
Data collection	The Pediatric Dengue Cohort Study (PDCS) is an ongoing open prospective cohort of 4,000 children 2-17 years old that was initiated in 2004. Blood samples are collected annually, and additional samples are collected from symptomatic suspected dengue cases at the acute (0-6 days post-onset of symptoms) and convalescent (14-21 days post-onset of symptoms) phase.
Outcomes	DENV infections are evaluated and recorded yearly by comparing healthy annual serum/plasma samples collected in two consecutive years side-by-side by DENV inhibition enzyme-linked immunosorbent assay (iELISA). A >=4-fold increase in DENV iELISA titer between annual samples is considered as indication of an infection. We selected 64 pre-inapparent and 63 pre-symptomatic infection plasma samples among DENV-immune participants who had experienced one or more prior DENV infections.<br>Symptomatic dengue cases were confirmed by detection of DENV RNA by RT-PCR, real-time RT-PCR and/or virus isolation in the acute-phase sample; seroconversion of DENV-specific immunoglobulin M antibodies in paired acute-and convalescent-phase samples; or a >=4-fold increase in antibody titer by iELISA between acute and convalescent sera. The serotype of symptomatic infections was determined by RT-PCR or real-time RT-PCR. Inapparent infections were identified with a >=4-fold increase in iELISA titer among participants who did not present with illness to the study Health Center between annual samplings. In a subset, the serotype responsible for the inapparent infections that occurred before 2016 was identified by neutralization assays (DENV1-4) and/or a multiplex Luminex-based assay (E domain III [EDIII] of DENV1-4 and ZIKV) by comparing the antibody profile in the annual samples collected before and after the infection.

Dual use research of concern

Policy information about dual use research of concern

Hazards

Could the accidental, deliberate or reckless misuse of agents or technologies generated in the work, or the application of information presented in the manuscript, pose a threat to:

No	Yes	
[x]	[ ]	Public health
[x]	[ ]	National security
[x]	[ ]	Crops and/or livestock
[x]	[ ]	Ecosystems
[x]	[ ]	Any other significant area

Experiments of concern

Does the work involve any of these experiments of concern:

No	Yes	
[x]	[ ]	Demonstrate how to render a vaccine ineffective
[x]	[ ]	Confer resistance to therapeutically useful antibiotics or antiviral agents
[x]	[ ]	Enhance the virulence of a pathogen or render a nonpathogen virulent
[x]	[ ]	Increase transmissibility of a pathogen
[x]	[ ]	Alter the host range of a pathogen
[x]	[ ]	Enable evasion of diagnostic/detection modalities
[x]	[ ]	Enable the weaponization of a biological agent or toxin
[x]	[ ]	Any other potentially harmful combination of experiments and agents

Plants

Seed stocks	n/a
Novel plant genotypes	n/a
Authentication	n/a

ChIP-seq

Data deposition

- [ ] Confirm that both raw and final processed data have been deposited in a public database such as GEO.
- [ ] Confirm that you have deposited or provided access to graph files (e.g. BED files) for the called peaks.

Data access links<br>*May remain private before publication.*	n/a
Files in database submission	n/a
Genome browser session<br>(e.g. UCSC)	n/a

Methodology

Replicates	n/a
Sequencing depth	n/a
Antibodies	n/a
Peak calling parameters	n/a
Data quality	n/a
Software	n/a

Flow Cytometry

Plots

Confirm that:

- [ ] The axis labels state the marker and fluorochrome used (e.g. CD4-FITC).
- [ ] The axis scales are clearly visible. Include numbers along axes only for bottom left plot of group (a 'group' is an analysis of identical markers).
- [ ] All plots are contour plots with outliers or pseudocolor plots.
- [ ] A numerical value for number of cells or percentage (with statistics) is provided.

Methodology

Sample preparation	n/a
Instrument	n/a
Software	n/a
Cell population abundance	n/a
Gating strategy	n/a

- [ ] Tick this box to confirm that a figure exemplifying the gating strategy is provided in the Supplementary Information.

Magnetic resonance imaging

Experimental design

Design type	n/a
Design specifications	n/a
Behavioral performance measures	n/a

Acquisition

Imaging type(s)	n/a
Field strength	n/a
Sequence & imaging parameters	n/a
Area of acquisition	n/a

Diffusion MRI [ ] Used [x] Not used

Preprocessing

Preprocessing software	n/a
Normalization	n/a
Normalization template	n/a
Noise and artifact removal	n/a
Volume censoring	n/a

Statistical modeling & inference

Model type and settings	n/a
Effect(s) tested	n/a

Specify type of analysis: [ ] Whole brain [ ] ROI-based [ ] Both

Statistic type for inference

(See Eklund et al. 2016)

n/a

Correction

n/a

Models & analysis

n/a	Involved in the study
☒	☐ Functional and/or effective connectivity
☒	☐ Graph analysis
☒	☐ Multivariate modeling or predictive analysis

Functional and/or effective connectivity

n/a

Graph analysis

n/a

Multivariate modeling and predictive analysis

n/a
